# Gastric Ulceration and Immune Suppression in Weaned Piglets Associated with Feed-Borne *Bacillus cereus* and *Aspergillus fumigatus*

**DOI:** 10.3390/toxins12110703

**Published:** 2020-11-07

**Authors:** Xiaohui Li, Qiang Li, Yihui Wang, Zhenhai Han, Guanggang Qu, Zhiqiang Shen, Shujian Huang, Cheng He

**Affiliations:** 1 College of Life Science and Engineering, Foshan University, Foshan 528531, China; lxh7061@163.com (X.L.); hanzhenhai@139.com (Z.H.); sjhuang.foshan@163.com (S.H.); 2 Key Lab of Animal Epidemiology and Zoonoses, College of Veterinary Medicine, China Agricultural University, Beijing 100193, China; lq2018@cau.edu.cn (Q.L.); yihuiwang@cau.edu.cn (Y.W.); 3 Binzhou Animal Science and Veterinary Medicine Academy of Shandong Province, Binzhou 256600, China; Guanggangqu@163.com (G.Q.); bzshenzq@163.com (Z.S.)

**Keywords:** *Bacillus cerues*, *Aspergillus fumigatus*, DON, gastric ulcerations, immune responses, piglets

## Abstract

As a multifactorial cause, gastric ulceration-mediated diarrhea is widely prevalent in the weaned piglets, impairing pig health and economic benefits. With full implementation of antibiotic stewardship programs in China, *Bacillus cereus* (*B. cereus*) and *Aspergillus fumigatus* (*A. fumigatus*) were identified frequently in porcine feedstuffs and feeds of the animal industry. Association between feed-borne *B. cereus* and frequent diarrhea remains unclear. In the present study, we conducted a survey of *B. cereus* and *A. fumigatus* from feeds and feedstuffs in pig farms during hot season. Interestingly, *B. cereus*, *B. subtilis*, *B. licheniformis* and *B. thuringinesis* were isolated and identified from piglets’ starter meals to sow feeds, accounting for 56.1%, 23.7%, 13.7% and 6.5%, respectively. Obviously, both *B. cereus* and *B. subtili* were dominant contaminants in the survey. In an in vitro study, Deoxynivalenol (DON) contents were determined in a dose-dependent manner post fermentation with *B. cereus* (405 and DawuC). Subsequently, 36 weaned piglets were randomly assigned to four groups and the piglets simultaneously received the combination of virulent *B. cereus* (Dawu C) and *A. fumigatus* while animals were inoculated with *B. cereus* (Dawu C), *A. fumigatus* or PBS as the control group. Clinically, piglets developed yellow diarrhea on day 5 and significant reductions of relative body weight were observed in the *B. cereus* group, and co-infection group. More importantly, IgG titers against *Classical swine fever virus* (CSFV) and Porcine epidemic diarrhea (PED) were reduced dramatically during 14-day observation in co-infection group, the *B. cereus* (Dawu C) group or the *A. fumigatus* group. However, lower Foot and mouth disease (FMD) -specific antibodies were reduced on day 7 compared to those of the control group. Additionally, lower lymphocyte proliferations were found in the *B. cereus* group and the co-infection group compared to the control group. Postmortem, higher lesions of gastric ulceration were observed in the *B. cereus* group and the co-infection group from day 7 to day 14 compared with those of the *A. fumigatus* group and the control group. Compared to the *A. fumigatus* group, higher DON contents were detected in the stomach inoculated with *B. cereus* and the co-infection with *A. fumigatus*. In conclusion, our data support the hypothesis that *B. cereus* might be associated with severe diarrhea by inducing gastric ulcerations and *A. fumigatus* might aggravate immune suppression, threating a sustainable swine industry. It is urgently needed to control feed-borne *B. cereus* contamination.

## 1. Introduction

Diarrhea induced by gastric ulceration has been observed frequently both in poultry flock and livestock in the past decades. Although gastric ulceration is associated with multiple factors, such as malnutrition, viral invasion, bacterial infection and mycotoxin contamination, mild or severe diarrhea is manifested as a clinical consequence, causing poor productivity, sudden death and economic losses for swine industry [1,2,3].

*Bacillus cerues* (*B. cereus*), an aerobic or facultative anaerobic bacteria that is spore-forming and that offers the ability to survive in most extreme conditions, spreads in the surroundings during agricultural/farming processes and can easily contaminate food and feed [4]. As a common opportunistic pathogen involved in food poisoning, *B. cereus* causes vomiting and diarrhea in humans frequently, which seriously leads to death [5,6]. In addition to gastrointestinal tract, *B. cereus* causes a number of systemic and local infections in both immunologically compromised and immunocompetent individuals [7]. Extensive research confirms that the intoxicated emetic syndrome is caused by an emetic toxin, termed cereulide, meanwhile the diarrheal syndrome is associated with pore-forming cytotoxins hemolysin HBL (*Hbl*), nonhaemolytic enterotoxin (*Nhe*) and cytotoxin K (*CytK*) [8]. Although it is known as a pathogen, *B. cereus* is licensed as a pesticide for agricultural plants and probiotics for animal additives. Particularly, *B. cereus* is reported to maintain the balance of gastrointestinal microflora, to improve nutrient utilization ratio and to protect healthy digestive function [9]. However, the lack of maximal tolerable limitation (MTL) of *B. cereus* in probiotic products facilities high contamination of *B.cereus* from animal feeds to human food chains [10,11]. *B. cereus* isolates in bedding, feces, feed, liquid manure and raw milk was found to be 93.3%, 78.9%, 41.2%, 100.0% and 9.8%, respectively, in 10 local dairy farms [12]. Milk-borne *B. cereus* might represent a potential hazard to consumers due to it being inactivated during milk manufacturing [12,13]. The national survey in China recently found that 33.7% of animal-used probiotics were contaminated with human-risk pathogens, and also human intestinal anthrax toxin gene *cya* was transmitted from a nearby chicken and fish farm [14]. Therefore, the virulent *Bacillus* spp and other emerging pathogens in animal-used probiotics pose an emerging threat to food safety.

*Aspergillus fumigatus* (*A. fumigatus*) is one of the most common species of *Aspergillus* and possesses the ability of a causative agent invasive pathogen, contributing to infectious risks, such as asthma, chronic pulmonary infection or toxic effects [15,16]. Also, *A. fumigatus* is reported to induce immunosuppression by inhibiting the proliferation of T and B cells, leading to secondary infection [17]. In the previous report, 63 isolates out of 105 feed were *A. fumigatus*, and the other isolates were 21 *A. niger* and 11 *A. candidus*, respectively, indicating that the *A. fumigatus* isolate was a dominant agent in poultry diets [18].

Regarding the wide prevalence of diarrhea in weaned piglets, the association between gastric ulceration and feed-borne *B. cereus* or *A. fumigatus* is unknown. Our hypothesis is that *A. fumigatus* aggravates gastric ulceration and the immune suppression triggered by feed-borne *B. cereus*. Our investigation will shed light on understanding the pathogenesis of gastric ulceration and maintaining a sustainable swine industry.

## 2. Results

### 2.1. B. cereus and B. subtilis Were Highly Contaminated in Swine Feed

During sampling period from June to August 2018, *B. cereus*, *B. subtilis*, *B. licheniformis* and *B. thuringinesis* were isolated from piglets’ starter meals to pregnant sow’s meals; 78 strains (56.1%) of *B. cereus*, 33 strains (23.7%) of *B. subtilis*, 19 strains (13.7%) of *B. licheniformis* and 9 strains (6.5%) of *B. thuringinesis* out of 139 isolates were identified by biochemical test and Polymerase chain reaction(PCR) assay. Obviously, both *B. cereus* and *B. subtilis* were dominant distributions in the swine feeds, accounting for 111 isolates (79.9%) (Table 1). Regarding the enumeration of *B. cereus*, 62.8% samples were found to contaminate with above 10,000 CFU/g, while 27.9% samples were arranged from 1000 to 10,000 CFU/g in swine meals (Table 2).

### 2.2. Increasing DON Production Post Fermentation with B. cereus Isolates

**Figure 1:**
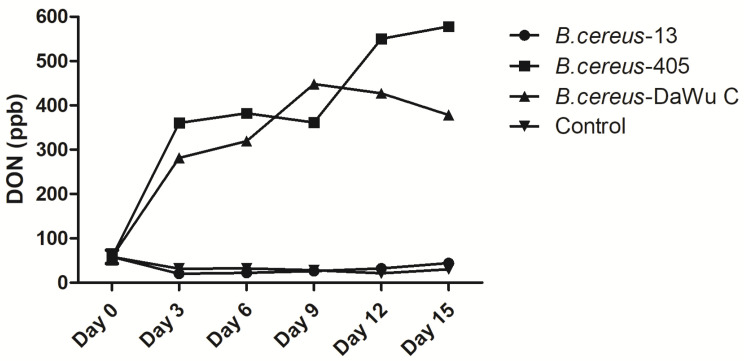
DON production in a dose-dependent manner post fermentation with *B. cereus* isolates for 15 days. *B. cereus*-13 and *B. cereus*-405 were isolated from pig meals, while *B. cereus* Dawu C was isolated from the lungs of the diseased layers. DON concentrations were determined by commercial ELISA kits (Beacon Analytical System Inc, Saco, ME, USA).

Both *B. cereus* strain (Dawu C) from the diseased layer and *B. cereus* isolate (405) from swine feeds produced DON in a dose-dependent manner from day 0 to day 9. Afterwards, DON productions were reduced gradually in the feeds fermented with the *B. cereus* isolate (DaWu C). However, the *B. cereus* isolate (13) from swine feedstuff yielded lowly to DON production and no difference was detected compared to the control group during the observation (Figure 1).

### 2.3. B. cereus, A. fumigatus and co-infection Exacerbated Diarrhea and Piglets’ Growth

**Figure 2:**
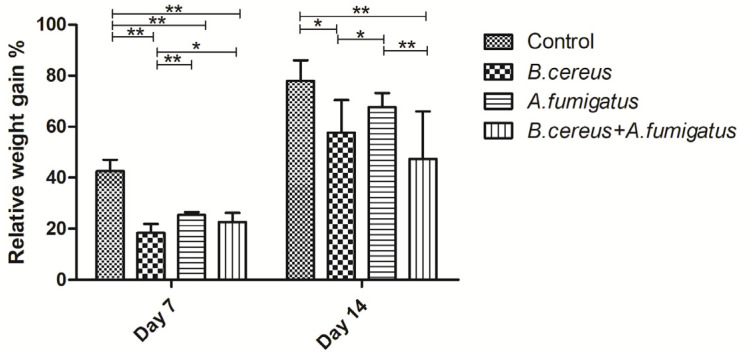
Relative body weight gains post inoculation with *B. cereus*, *A. fumigatus* or the combination. Average relative body weight gain was significantly reduced in the all-inoculated groups compared to the control group from day 7 to day 14. Compared to the *A. fumigatus* group, the *B. cereus* group preferred to induce lower body weight gain on both day 7 and day 14 while the co-infection group induced a significant reduction of body weight gain on day 14. The data were expressed as the mean ± SD (*n* = 9; *n* = 6). *: *p* < 0.05; **: *p* < 0.01.

After inoculation with different pathogens, 3 piglets from the *B. cereus* group and 2 piglets from the co-infection group developed mild diarrhea on day 5. Afterwards, 5 piglets and 2 animals from above two groups were observed to have severe diarrhea with poor activity and low appetite. However, no mortality occurred until last observation in all the groups. As for body weight, no difference of body weight was found among the groups before treatment. On day 7, the relative average body weight gain was reduced extremely significantly in the *B. cereus* group (*p* < 0.01), *A. fumigatus* group (*p* < 0.01) and co-infection group (*p* < 0.01) compared to the control group. Later on, the significant reduction of body weight gain was observed in the *B. cereus* group (*p* < 0.05) and co-infection group (*p* < 0.01) compared to the control group or the *A. fumigatus* group. Also, the co-infection group exhibited a significant decrease compared to the control group or the *A. fumigatus* group on day 14 (*p* < 0.01) (Figure 2).

### 2.4. B. cereus and A. fumigatus Reduced CSFV-, FMD- and PED-Specific Antibodies

**Figure 3:**
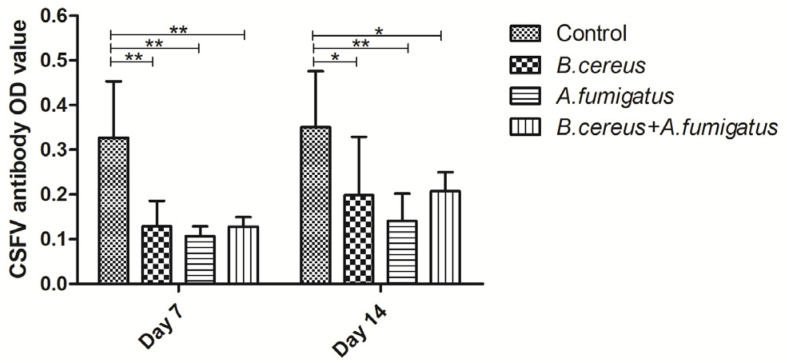
Detecting *Classical swine fever virus* (CSFV)-specific antibodies post inoculation with *B. cereus*, *A. fumigatus* or the combination of *B. cereus* and *A. fumigatus*. Compared to the control group, CSFV antibodies were reduced dramatically in the *B. cereus* group, the *A. fumigatus* group and the co-infection group from day 7 (*p* < 0.01) to day 14 (*p* < 0.05). No significant difference was found among the *B. cereus* group, the *A. fumigatus* group and the co-infection group from day 7 to day 14. The data were expressed as the mean ± SD (*n* = 9; *n* = 6). *: *p* < 0.05; **: *p* < 0.01.

**Figure 4:**
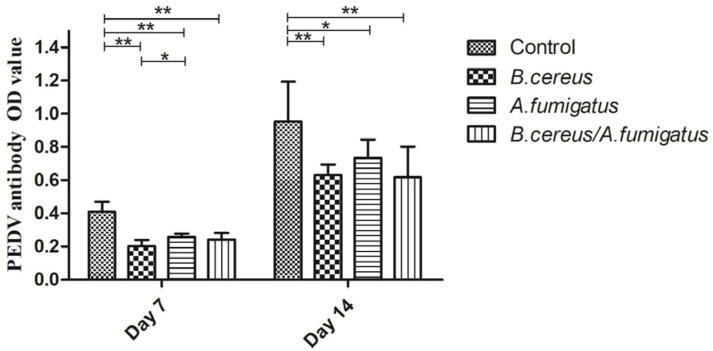
Detecting Porcine epidemic diarrhea (PED)-specific antibodies post inoculation with *B. cereus*, *A. fumigatus* or the combination of *B. cereus* and *A. fumigatus*. In comparison with the control group, lower PED-specific IgG antibodies were detected in the *B. cereus* group, the *A. fumigatus* group and the co-infection group than those of the control group from day 7 (*p* < 0.01) to day 14 (*p* < 0.05). A significant difference was found between the *B. cereus* group and the *A. fumigatus* group on day 7 (*p* < 0.05), and no statistic difference was found among the *B. cereus* group, the *A. fumigatus* group and the co-infection group on day 14. The data were expressed as the mean ± SD (*n* = 9; *n* = 6). *: *p* < 0.05; **: *p* < 0.01.

**Figure 5:**
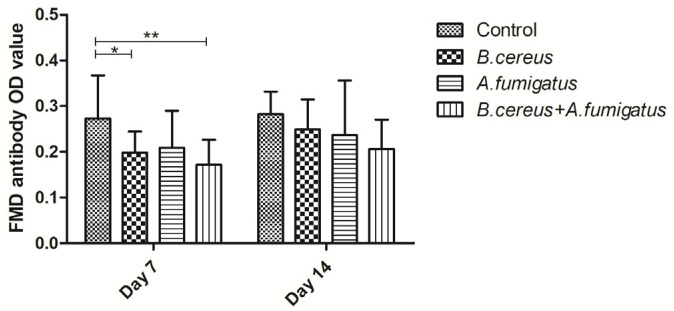
Detecting Foot and mouth disease (FMD)-specific antibodies post inoculation with *B. cereus*, *A. fumigatus* or the combination of *B. cereus* and *A. fumigatus*. As for FMD-specific antibodies, a statistically significant decline was found in the *B. cereus* group compared to the control group on day 7 (*p* < 0.05). However, no statistical difference was found among three treated groups on day 14. The data were expressed as the mean ± SD (*n* = 9; *n* = 6). *: *p* < 0.05; **: *p* < 0.01.

Compared to the control group, *Classical swine fever virus* (CSFV) antibodies were dramatically reduced in the *B. cereus* group, the *A. fumigatus* group and the co-infection group from day 7 (*p* < 0.01) to day 14 (*p* < 0.05). Afterwards, obviously lower CSFV antibodies were found in the *A. fumigatus* group compared to the other groups on day 14 (*p* < 0.01), but no statistical difference was detected among the *B. cereus* group and the co-infected group at the two time points (Figure 3). Similarly, lower Porcine epidemic diarrhea (PED)-specific immunoglobulin G (IgG) antibodies were detected in all treatment groups compared to the control group from day 7 (*p* < 0.01) to day 14 (*p* < 0.05). However, no statistical difference was detected between the *B. cereus* group and the co-infection group on day 14. Thus, no statistic difference was found among the *B. cereus* group, the *A. fumigatus* group and the co-infection group on day 14 (Figure 4). As for Foot and mouth disease (FMD)-specific antibodies, a statistically significant decline of FMD specific antibodies in the *B. cereus* group (*p* < 0.05) and the co-infection group(*p* < 0.01) was compared to that of the control group on day 7 and no statistical difference was found on day 14 (Figure 5). A statistically significant decline of FMD specific antibodies in the *B. cereus* group and the co-infection group was compared to the control group on day 7 (*p* < 0.05) and no statistical difference was found on day 14 (Figure 5).

### 2.5. B. cereus and Co-Infection Reduced Lymphocyte Proliferation

**Figure 6:**
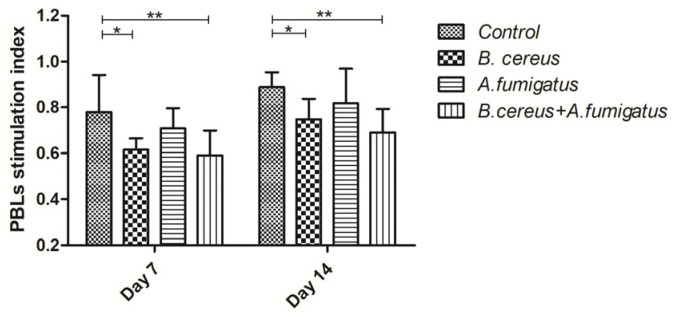
Stimulation index of peripheral blood lymphocyte (PBL) post inoculation with *B. cereus*, or *A. fumigatus* or the combination of *B. cereus* and *A. fumigatus*. Lower proliferations were determined in the *B. cereus* group (*p* < 0.05) and the co-infection group (*p* < 0.01) compared to the control group from day 7 to day 14. The data were expressed as the mean ± SD.*: *p <* 0.05; **: *p <* 0.01.

For the lymphocyte stimulation level, there was a comparable trend in all group at two points. Lower proliferations were determined in the *B. cereus* group (*p* < 0.05) and co-infection group (*p* < 0.01) compared to the control group from day 7 to day 14. However, no significant difference was found among *B. cereus* group, *A. fumigatus* group and co-infection group (Figure 6).

### 2.6. Both B. cereus and co-infection Induced Typic Gastric Ulcerations in Piglets

**Figure 7:**
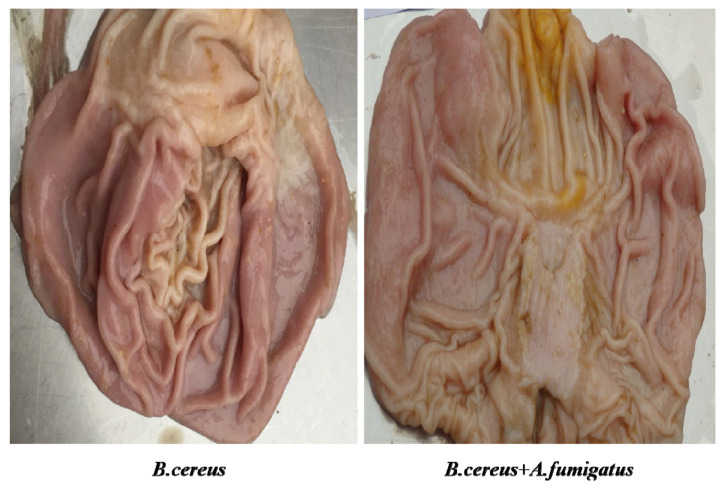
Gastric lesions post inoculation with *B. cereus, A. fumigatus* or the combination of *B. cereus* and *A. fumigatus* on day 7. Gastric ulcerations were observed both in the *B. cereus* group and the co-infection group. Severe ulcerations were evident in the *B. cereus* group on day 7.

**Figure 8:**
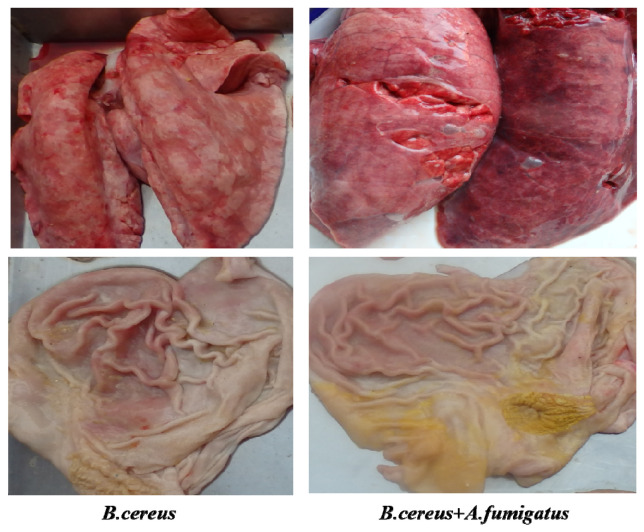
Gastric lesions and hemorrhagic lungs post inoculation with *B. cereus, A. fumigatus* or the combination of *B. cereus* and *A. fumigatus* on day 14. Gastric ulcerations and hemorrhagic inflammations were observed both in the *B. cereus* group and the co-infection group, but severe hemorrhagic lungs were developed in the co-infection group on day 14.

**Figure 9:**
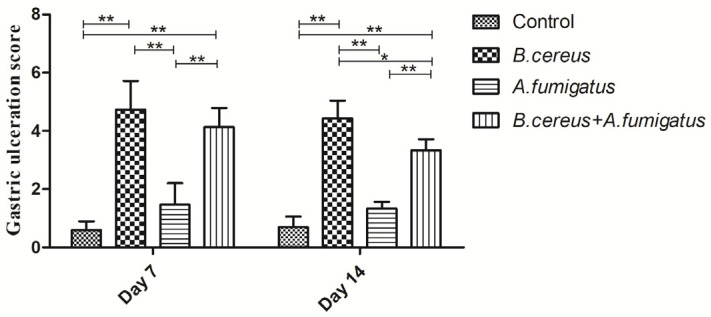
Lesion scores of gastric ulcers post inoculation with *B. cereus, A. fumigatus* or the combination *B. cereus* and *A. fumigatus*. Postmortem on day 7 and day 14, typic gastric ulceration was observed both in the *B. cereus* group and the co-infection group. Severe lesions of gastric ulcerations were found in the *B. cereus* group (*p <* 0.01) and the co-infection group (*p <* 0.01) compared to the control group and the *A. fumigatus* group at two-time points. The data were expressed as the mean ± SD (*n* = 9; *n* = 6). *: *p <* 0.05; **: *p <* 0.01.

In the postmortem on day 7 (*n* = 3), typic gastric ulceration and hemorrhagic erosions were observed both in the *B. cereus* group and the co-infection group (Figure 7). Later on, severe gastric erosions and hemorrhagic inflammations in the lungs were observed in both the *B. cereus* group and the co-infection group, but massive hemorrhagic lungs were characterized in the co-infection group (Figure 8). The higher lesions of gastric ulcerations were found in the *B. cereus* group and the co-infection group compared to the *A. fumigatus* group and the control group (Figure 9). The remaining 6 piglets developed severe gastric ulcerations with hemorrhagic lungs in the co-infection group, while 3 out of 6 piglets were observed with gastric ulcerations and 1 piglet developed hemorrhagic ulceration in the *B. cereus* group.

### 2.7. B. cereus and co-infection Induced DON Production in Piglets’ Gastric Ulcers

**Figure 10:**
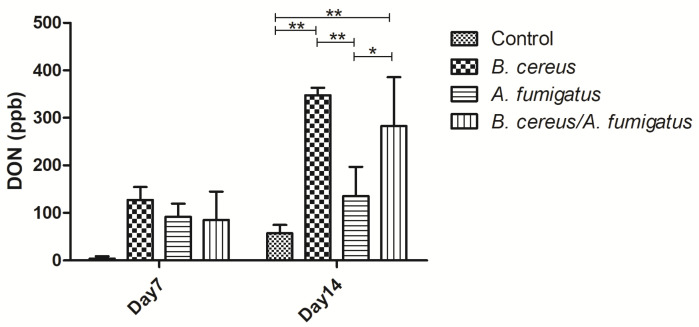
*B. cereus*, *A. fumigatus* and co-infection induced DON production in piglets’ gastric ulcers. Lower DON contents were determined among the *B. cereus* group, *A. fumigatus* group and co-infection group on day 7. Later on, higher DON productions were detected in the *B. cereus* group (*p* < 0.01) and co-infection group (*p* < 0.01) compared to the control group. Compared to the *A. fumigatus* group, significant increasing DON contents were found in the *B. cereus* group (*p* < 0.01) and co-infection group (*p* < 0.05). The data were expressed as the mean ± SD (*n* = 9; *n* = 6). *: *p <* 0.05; **: *p <* 0.01.

On day 7, lower DON contents were determined among *B. cereus* group, *A. fumigatus* group and co-infection group. However, no significant difference was found compared to the control group. Later on, higher DON contents were detected in the *B. cereus* group (*p* < 0.01) and the co-infection group (*p* < 0.01) compared to the control group on day 14. Compared to the *A. fumigatus* group, significant increasing DON contents were found in the *B. cereus* group (*p* < 0.01) and co-infection group (*p* < 0.05) (Figure 10).

## 3. Discussion

Ulceration of the stomach is a common disease, amounting to 93% prevalence in the pig industry. The exact mechanism of gastric ulcers remains unclear due to multifactorial agents, such as diet size, management and infectious agents. Severe respiratory infections have been associated with gastric lesions, such as porcine reproductive and respiratory syndrome (PRRS), post-weaning multisystemic wasting syndrome (PMWS), swine influenza, *Actinobacillus pleuropneumoniae* or porcine circovirus type 2, and the *Ascaris suum* infestation or Mycoplasma vaccination [3,20]. *Lactobacillus*-elicited score was significantly greater in the antrum and corpus of stomachs without ulcers when compared with stomachs with ulcers in gnotobiotic pigs [21]. *Helicobacter suis* (*H. suis*) has been associated with development of gastric ulcers in the non-glandular part of the porcine stomach by decreasing the *H. suis*-binding ability of the mucins and impairing the mucus barrier. More recently, *Fusobacterium gastrosuis* (*F. gastrosuis*) is identified based on 16S rRNA and *gyrase B* genes, and it was hypothesized that this micro-organism could play a role in the development of gastric ulceration because most *Fusobacterium* spp can aggravate necrosis [22]. *H. suis*-infected pigs showed a significantly higher colonization rate of *F. gastrosuis* in the non-glandular gastric region compared to non-infected pigs [23]. A previous study has demonstrated an association between gastric lesions and a carbohydrate-enriched liquid diet when mono-infected with *Lactobacillus sp.* and *Bacillus sp.*, from which the source of these contaminations was not determined [24]. However, association of feed-borne *B. cereus* and gastric ulcerations has not been illustrated so far.

In the present study, both *B. cereus* and *B. subtilis* contamination were dominated in pig meals, while 62.8% feed samples accounted for 10,000 colonies of *B. cereus,* and *B. cereus* contamination would amount to 3 × 10^6^ CFU/g (average 300 g feed intake per day). Moreover, DON production showed a dose-dependent manner post fermentation with two *B. cereus* isolates, contributing to one of main resources of DON contamination in animal feed. In vivo study, the animal’s relative body weight gain, lymphocyte proliferations and antibodies against CSFV, FMD and PED were reduced significantly post inoculation with *B. cereus, A. fumigatus* or combination of two pathogens. More important, typic diarrhea and gastric ulcerations were observed in the piglets that received *B. cereus* or combination of *B. cereus* and *A. fumigatus*. Later on, both increasing gastric ulceration and hemorrhagic inflammations in the lungs were observed postmortem in comparison with the *A. fumigatus* group and the control group. Our study supports our hypothesis that feed-borne *B. cereus* triggers diarrhea and respiratory distress by inducing gastric ulceration and hemorrhagic lesions in the lungs. Afterwards, the secondary *A. fumigatus* infection and DON poisoning exacerbate clinical maldigestion and vaccine failure, leading to a high risk for the piglet’s survival and growth.

Although typic gastric ulcerations were evident both in the piglets who received *B. cereus* or a combination of *B. cereus* and *A. fumigatus*, the correlation between *B. cereus* and the development of gastric ulcers remains unclear. In our preliminary investigation, piglets’ diarrhea is prevalent in the hot season from June to September in Northern China. Particularly, increasing gastric ulcers are reported in pig farms in summer. In this sense, gastric ulcerations are associated with hot climate. In early infection, *B. cereus* may adhere to keratinized epithelia of the pars esophagea, contributing to total gastric acid concentration by the production and release of lipid-soluble acidic metabolites such as lactic, acetic and propionic acids, ethanol and hydrogen peroxide. Piglets experimentally infected with *B. cereus* were reduced in body weight gain due to pain and inflammation in stomach, leading to a more fluid gastric content, breakdown of the pH gradient and irritation of the pars oesophagea [25]. Erosions and gastroesophageal ulcers were observed in the pars esophagea of young gnotobiotic swine fed a carbohydrate-enriched liquid diet and mono-infected with *Lactobacillus sp.* and *Bacillus sp.* [24]. In our previous study, feed-borne *B. cereus* co-infection with avian influenza virus (H9N2) has produced significant gizzard erosions and ulceration(GEU) in all bird groups by damaging to the epithelium of the digestive tract, which facilitates other susceptible pathogens [19]. More recently, chickens exposed to the *B. cereus* co-infection with *Chlamydia psittaci* developed a severe GEU syndrome suggesting that injury to the koilin layer of the gizzard with *B. cereus* toxins could directly affect the gizzard membrane, while the intraesophageal *C.* *psittaci* infection also promotes the development of the GEU [26]. These data suggest that feed-borne *B. cereus* is an important interactive factor for development of GEU in piglets. In addition, several toxic genes, such as *nhA*, *nhB*, *nhC*, *Hbl* and *Cytk*, were identified in *B. cereus* (Dawu-C) [26], which may contribute to the diarrhea by disrupting the epithelial layer [27]. Some of these toxins appear to play a major role in pathogenesis during *B. cereus* gastroenteritis and opportunistic infections, although no direct connection has been shown. After fermentation with the *B. cereus* Dawu C strain, DON production was in a dose-dependent manner within two weeks, leading to long term diarrhea by inducing pores in epithelial cells, necrosis and mucosal damage. In time, significant increasing DON contents were determined both in the piglets with *B. cereus* or a combination of *B. cereus* and *A. fumigatus*, indicating that the combination of *B. cereus* and DON might contribute to the development of gastric lesions. DON is reported to reduce body weight, feed conversion and immunosuppression by inhibiting protein synthesis, disrupting signal transmission and eventually causing cell death [28].

Regarding immunosuppression post inoculation with *B. cereus* or a combination of *B. cereus* and *A. fumigatus*, it might be associated with dysfunction of macrophages post infection with *B. cereus*. *B. cereus* can escape immune surveillance and transport through the whole body with macrophages by impairing innate immunity, leading to immune inhibition [29]. On other hand, *A. fumigatus* is the predominant mold agent of the immunosuppressed animals. Once the immune system is comprised, the fungus conidia is able to germinate into hyphae and establish a focal infection within lungs. Also, *A. fumigatus* is involved in disrupting the antigen processing and inhibiting the proliferation of T and B cells [17,30]. In the study, co-inoculation with *B. cereus* and *A. fumigatus* reduced the humoral immune response, characterized as lower lymphocyte proliferation and poor antibodies against CSFV or FMD and PED. More important, the *B. cereus* co-infection with *A. fumigatus* contributed to severe hemorrhagic inflammations in the lungs except for gastric ulcerations in piglets.

## 4. Conclusions

In conclusion, our pioneer study indicated that birds orally administered *B. cereus* exhibited the GEU and hemorrhagic inflammations in the lungs of chickens. In the present study, feed-borne *B. cereus* initiated diarrhea by inducing gastric ulceration and hemorrhagic lungs, and secondary *A. fumigatus* infections exacerbated gastric lesions and immunosuppression by inducing low lymphocyte proliferation and poor humoral immune response. Therefore, feed-borne *B. cereus* and its secondary metabolites are urgently needed for further investigation for combating the high incidence of diarrhea and for maintaining a sustainable porcine industry. Most important, it is the first report that gastric ulceration has been induced by *B. cereus* alone in piglets and it might be a good animal for understanding human gastric ulceration.

## 5. Materials and Methods

### 5.1. Bacillus Spp Isolation, Identification and Contamination Status

Both *B. cereus* and *A. fumigatus* used in the trial were isolated from feeds and feedstuffs in breeding pig flock (Daxing, Beijing, China). Pellets or powders (10 g) were mixed with sterile distilled water (90 mL) and plated for enumeration. The most probable number (MPN) method was recommended for routine surveillance of swine feeds and feedstuffs. Briefly, samples were inoculated into trypticase soy-polymyxin broth by preparation of 10^−1^, 10^−2^ and 10^−3^ dilutions. The tubes were incubated for 48 ± 2 h at 30 °C and observed for turbid growth. Afterwards, positive tubes were streaked onto Mannitol Yolk Polymyxine Agar (MYP) (Oxoid, Beijing, China), and then colonies were grown for 18–24 h at 30 °C. Typical colonies grown on MYP were confirmed with a biochemical test as described previously [31]. Furthermore, isolates of *Bacillus* spp were identified by molecular analysis using the 16s rRNA gene, *nheA* gene, *nheB* gene, *nheC* gene, *Em1*gene and *CytK* gene as previously described [19]. The MPN of *B. cereus* was detected based on the number of tubes at each dilution in which the presence of *B. cereus* was noted [32]. Meanwhile, the same diet was diluted to grow onto the Sabouraud dextrose agar (SDA) (Oxoid, Beijing, China) following the protocol, and identification was confirmed by PCR [18].

### 5.2. Determination of Deoxynivalenol (DON) Post Fermentation with Bacillus cereus Isolates

Roughly 1000 g of feeds were pretreated with Co60 to avoid additional microbial contamination and to ensure low DON contents (less than 100 ppb); then the samples were mixed with 3 *B. cereus* isolates (405, Dawa C, 13) and fermented at 25 °C and 60% humidity for two weeks. After fermentation, 100 g feed samples were collected on day 0, 3, 6, 9, 12 and 15, respectively, and DON concentrations were determined by the DON plate kit following the protocols (Beacon Analytical System Inc).

### 5.3. Animals and Ethics Statement

The weaned piglets aged 32 days were purchased from the Daxing Breeder Animal Company, Beijing, China. All the animals were given food and water ad libitum. The experimental protocols were approved by an Ethical Reviewing Board at China Agricultural University on Institutional Animal Care and Use Committee(IACUC) (code: IACUC20170701). The date of approval was 1 July 2017 by IACUC at CAU. This protocol follows humane protocols that minimize pain in the animals. Briefly, any potential pain, distress or discomfort should be minimized or alleviated by choosing the earliest endpoint that is compatible with the scientific objectives of the research. Selection of this endpoint should involve consultation with the laboratory animal veterinarian and the animal care committee [33]. Prior to treatment, all suckling piglets received a vaccination program, such as the attenuated vaccine against *Classic swine fever virus* (CSFV) on day 1, the live vaccine against Porcine epidemic diarrhea (PED) on day 7 and the inactivated vaccine against Foot and mouth disease (FMD) on day 30. Thirty six weaned piglets were randomly assigned to four groups, and Group 1 animals were inoculated with PBS daily as a negative control. Group 2 piglets received orally 1 × 10^8^ MPN/mL of *B. cereus* for 14 days, and Group 3 animals were administered orally 1 × 10^8^ CFU/mL of *A. fumigatus* for 14 days. Group 4 piglets were orally inoculated with 1 × 10^8^ MPN/mL of *B. cereus* and 1 × 10^8^ CFU/mL of *A. fumigatus* at the same time and this lasted for 14 days. All the animals were raised separately and fed twice a day with commercial pellet diets and had access to drinking water freely all day. All groups were weighed weekly. Clinical signs were recorded a minimum of twice daily for 14 days, including depression, inappetence, coughing, respiratory distress and diarrhea.

### 5.4. Detecting Antibodies against CSFV, PED and FMD

Serum samples were collected by venipuncture before inoculation (9 animals per group) on day 7 (9 animals per group) and day 14 (6 piglets per group). The sera were prepared by centrifuging at 3500 rpm/min for 10 min and stored at −20 °C until use. CSFV-specific antibodies, PEDV-specific antibodies and FMD-specific antibodies were measured using the specific commercial kit (IDEXX, Beijing, China) according to the manufacturer’s protocol.

### 5.5. Lymphocyte Proliferation Assay

Peripheral blood mononuclear cells (PBMCs) were prepared, and lymphocyte proliferation was determined using the BrdU Cell Proliferation ELISA Kit (Abcam, Beijing, China). Briefly, PBMCs were stimulated with inactivated whole *B. cereus* at 5 µg/well as specific antigens while Concanavalin A (ConA) (Sigma-Aldrich, Saint Louis, MS, USA) was added at 5 μg/well as a positive control and the medium was used as a negative control. All experiments were performed in triplicate following the manufacturer’s instructions. Results were expressed as the stimulation index (SI), calculated as the mean of the stimulation index for antigen-stimulated wells divided by the stimulation index for medium control wells with the background subtracted.

### 5.6. Pathological Evaluation

Piglets were sacrificed on day 7 (3 animals per group) and day 14 (6 animals per group) post inoculation. The stomachs were exteriorized, ligated at the esophagus and duodenum, removed and aseptically opened along the greater and less curvatures. Each stomach was dissected along the large curvature and all of the gastric contents were removed at room temperature. Gastric ulcers were assessed immediately on a scale between 1 and 9 [34] (1 = normal stomach; 2 = slight hyperkeratosis of stomach epithelium; 3 = moderate hyperkeratosis; 4 = severe hyperkeratosis; 5 = erosions and ulcer covering less than 2 cm^2^; 6 = ulcer 2–8 cm^2^; 7 = ulcer 8–16 cm^2^; 8 = ulcer greater than 16 cm^2^ and scar; 9 = death due to ulcer). Meanwhile, occurrence rates of ulceration were summed up in accordance with the relative area.

### 5.7. Quantitative DON in Stomach

Gastric contents were collected from the distal esophagus and pars esophagea, and roughly 10 g of contents were homogenized with 200 mL deionized water and then centrifugated at 4000 rpm/min for 5 min. Finally, 5 mL supernatants were collected for DON testing using the above ELISA kit (Beacon Analytical System Inc., Saco, ME, USA).

### 5.8. Statistical Analysis

Relative weight gain, antibody levels, lymphocyte proliferation and lesion evaluation were statistically analyzed using SPSS 25.0 version to perform the one-way ANOVA with the Least Significance Difference (LSD) post hoc test on at least three independent replicates. *p*-values of <0.05 were considered statistically significant for each test, and when *p* < 0.01, the results were extremely significant.

## Figures and Tables

**Table 1:** Positive *Bacillus* spp were isolated and identified in feed and feed stuff in pig meals.

Feedstuffs	Samples	Positive			
		*B. cereus*	*B. subtitis*	*B. licheniformis*	*B. thuringiensis*
Started meals	34	18(52.9)	9 (26.5)	5 (14.7)	2 (5.9)
Growing meals	36	23 (63.9)	8 (22.2)	3 (8.3)	2 (5.6)
Finished meals	35	22(62.9)	6 (17.1)	4 (11.4)	3 (8.6)
Sow’s meals	34	15 (44.1)	10 (29.4)	7 (20.6)	2 (5.9)
Total	139	78 (56.1)	33 (23.7)	19 (13.7)	9 (6.5)

Notes: 139 samples were collected during June to August 2018 in summer in Daxing District, Beijing, China. *B. cereus*, *B. subtitis*, *B. licheniformis* and *B. thuringiensis* were identified by biochemical test and Polymerase chain reaction(PCR) assay [19].

**Table 2:** Enumeration of *B. cereus* in porcine meals.

Feedstuffs	Samples	*B. cereus* (CFU/g)			
		<10	10–1000	1000–10,000	>10^4^
Started meals	10	1 (10.0)	1 (10.0)	3 (30.0)	6 (60.0)
Growing meals	11	0 (0.0)	1 (9.1)	3 (27.3)	7 (63.6)
Finished meals	12	0 (0.0)	1 (8.3)	3 (25.0)	8 (66.7)
Sow meals	10	1 (2.3)	1 (10.0)	3 (30.0)	6 (60.0)
Totals	43	1 (2.3)	4 (9.3)	12 (27.9)	27 (62.8)

Notes: Conies of *B. cereus* determined on Mannitol Yolk Polymyxine (MYP) agar after serial dilutions and inoculation at 30 °C.
